# Effects of Adrenocorticotropic Hormone Treatment on Heart Muscle in Patients with Infantile Spasm

**DOI:** 10.7759/cureus.7121

**Published:** 2020-02-27

**Authors:** Gültekin Kutluk, Filiz Ekici, Özlem Turan, Ömer Bektaş, Naz Kadem

**Affiliations:** 1 Pediatric Neurology, Antalya Research and Training Hospital, Antalya, TUR; 2 Pediatric Cardiology, Akdeniz University, Faculty of Medicine, Antalya, TUR; 3 Pediatric Cardiology, Antalya Research and Training Hospital, Antalya, TUR; 4 Pediatric Neurology, Ankara University, Faculty of Medicine, Ankara, TUR; 5 Pediatrics, Antalya Research and Training Hospital, Antalya, TUR

**Keywords:** infantile spasm, adrenocorticotropic hormone, systolic and diastolic myocardial functions, children, cardiovascular side effects

## Abstract

Background and aim

Infantile spasm (IS) is a common epileptic syndrome of childhood epilepsies. The most effective treatment for IS is adrenocorticotropic hormone (ACTH). We hypothesized that ACTH treatment might change myocardial systolic and diastolic performance and cause cardiovascular side effects. This study aims to evaluate the effects of ACTH treatment on the heart muscle in IS patients.

Materials and methods

Eighteen newly diagnosed patients with IS participated in the study. ACTH (Synacthen® Depot) administered for two months in a total of 18 doses. A twelve-channel-surface electrocardiogram (ECG) and echocardiography performed in all patients before ACTH treatment, the second month after ACTH treatment (end of treatment), and two months later (after treatment). The systolic and diastolic myocardial functions were assessed by conventional and tissue Doppler imaging (TDI).

Results

The mean age of the patients was 8.1 months, and the patient group consisted of five female and 13 male subjects. None of the patients had clinically significant arrhythmia during treatment. After treatment, the mean heart rates of the patients significantly decreased (p=0.02), the systolic and diastolic blood pressures of patients did not change. We observed mild septal hypertrophy and an increase in the left ventricle mass index with ACTH treatment. Septal hypertrophy did not show progression until the fourth month after treatment. After ACTH treatment, patients had higher left ventricular myocardial performance index and lower E′ and A′ values at the mitral lateral annuli, however, these values didn't statistically significant from pretreatment values.

Conclusion

The low dose and short duration ACTH treatment in IS patients may cause subclinical myocardial hypertrophy. ACTH treatment has no significant side effects on cardiac functions.

## Introduction

Infantile spasm (IS) / West syndrome is an epileptic syndrome seen in the early childhood period that is characterized by tonic spasms and hypsarrhythmia on electroencephalography (EEG). It comprises 7.5-20% of childhood epilepsies [1, 2]. Since 1958, the most effective treatment method for IS is adrenocorticotropic hormone (ACTH) and steroids. The mechanism of ACTH on IS is not precise, but it is considered to cause reductions in stress hormone and corticotropin-releasing hormone release. Besides its high efficacy, it has significant systemic and cardiovascular side effects [3, 4]. We hypothesized that ACTH treatment might change myocardial systolic and diastolic performance and cause clinical/subclinical cardiovascular side effects. In this study, we aimed to evaluate the effects of ACTH treatment on the heart muscle in IS patients.

## Materials and methods

Study group

Infants who were under 24 months of age, and consecutively diagnosed with IS in our pediatric neurology department were included in this study. Twenty four newly diagnosed patients with infantile spasm participated in the study before ACTH treatment. Before diagnosis, at least one 21 channel electroencephalogram (EEG) recording was performed on all patients during stage 2 sleep states. Electrodes were placed using the International 10-20 System with bipolar and referential montages (including the Oz electrode). Interelectrode resistance was below 3 kΩ, and filters were set at 0.3 to 70 Hz. All patients had hypsarrhythmia on EEG according to criteria defined by Gibbs and Gibbs [5]. Two patients were excluded due to treatment incompliance, two patients' treatment changed due to lack of response, and two patients died after a short period after beginning treatment. Written informed consent was obtained from all parents of the children, which was approved by our Hospital Ethics Committee (Date: 30.06.2009 number: 14). 

The percentile intervals for patients were assessed according to height and weight. A 12 channel electrocardiograph (ECG) and echocardiography performed on all patients before ACTH treatment. All participants were free of any cardiac abnormalities. Cases with congenital heart disease, lung diseases, maternal diabetes history, history of using steroids or other medications before diagnosis, metabolic or genetic diseases, family history of cardiomyopathy, and other systemic diseases like hypertension were excluded from the study.

ACTH (Synacthen® Depot) administered for two months in a total of 18 doses (0.5 mg/kg < 10 kg; 1 mg/kg >10 kg). 

Echocardiographic examinations 

Echocardiographic examinations were performed at the beginning of the treatment (before treatment). All patients were reevaluated by echocardiography in the second month (end of treatment) and two months later (after treatment).

Standard echocardiography and TDI studies were performed in all patients by one pediatric cardiologist who was blinded to the clinical details and by using Vivid 7 Pro (3 MHz transducers; GE, Horten, Norway) echocardiography device. Two-dimensional, M-mode, pulsed-wave (PW), and continuous-wave Doppler echocardiographic images were obtained. Shortening fraction (SF) of the left ventricle (LV) were calculated from M-mode measurements of LV dimensions at the level of mitral valve leaflets in the parasternal long-axis view. The sample volume of the PW Doppler was placed between the tips of the mitral leaflets in the apical four-chamber view, and then diastolic functions of the left ventricles were measured (peak early [E] and late [A] wave velocities of the mitral valves as well as E/A ratio). The same echocardiography device performed TDI studies by switching to PW TDI mode. Apical four-chamber views were obtained and longitudinal peak annular velocities during systole (S'), early (E') and late diastole (A'), and the E'/A' ratio, deceleration time (DT), isovolumic contraction time (IVCT), isovolumic relaxation time (IVRT), and ejection time (ET) were measured at lateral annuli of the mitral valves. Myocardial performance index (MPI: IVRT+ICRT/ ET) was measured at the lateral annuli of the mitral valves [6].

Statistical analysis 

All statistical analyses were conducted using the Statistical Package for the Social Sciences (SPSS, Chicago, IL, USA), version 18.0 and Duncan test was performed with MSTAT-C program (Michigan State University, Michigan, USA), data are presented as the mean and standard deviation. Repeated measurements of all parameters were assessed with the variance analysis technique, while differences between the means were assessed with Duncan multiple comparison tests (0, 1, 2). Assessment of categoric variables used the Pearson chi-square test. A p-value 0.05 was considered statistically significant. 

## Results

The baseline demographic and clinical characteristics of the study subjects were presented in Table 1. The mean and median ages of the patients were 8.1 months and 7.3 months, respectively. The patient group consisted of five females (27.7%) and 13 male (72.2%) subjects.

**Table 1:** Demographic characteristics of patients SD - standard deviation

Demographic data	Patient group (n=18)
Age at diagnosis (months) Mean + SD range	8.1 ± 3.6 3 - 21
Gender: male / female	13 / 5
Weight (kg) mean + SD	7.8 ± 0.4
Height (cm) mean + SD	68.3 ± 1.4
Body surface area (kg/m^2^ ) mean + SD	0.38 ± 0.01

None of the patients had clinically significant arrhythmia during treatment. The mean heart rates at the end of the treatment and after the treatment were determined significantly decrease compared to before ACTH treatment (p=0.02 and p=0.02, respectively). Mean heart rate after treatment was not observed to be statistically significantly different compared to mean heart rate during treatment (p>0.05) (Table 2). When the systolic and diastolic blood pressure of patients before, end, and after treatment were compared, there was no statistically significant difference identified (p>0.05) (Table 2).

**Table 2:** Comparison of heart rate and blood pressure values of patients Values are expressed as mean ± standard deviation p^1^: comparison of before and the end of treatment (the second month). p^2^: comparison of before and after treatment (the fourth month). p^3^: comparison of the second and the fourth months of the treatment.

	Before treatment	The end of treatment (at the 2^nd^ month)	After treatment (at the 4^th^ month)	p^1^	p^2^	p^3^
Heart rate (beat/min)	131.5 ± 1.4	126.3 ± 1.7	123.4 ± 1.6	0.02	0.02	>0.05
Systolic blood pressure (mmHg)	94.1 ± 1.3	94.1 ± 1.4	93.3 ± 1.3	0.99	0.95	0.97
Diastolic blood pressure (mmHg)	51.4 ± 1.9	49.9 ± 1.5	53.7 ± 1.7	0.11	0.11	0.12

The interventricular septum systolic (IVSs) and diastolic (IVSd) thickness measured before treatment were determined to be statistically significantly increased compared to values at the end of the treatment. (p<0.01) (Table 3). Similarly, IVSs and IVSd values before treatment were identified to a statistically significant increase compared to values after the treatment (p<0.01). The left ventricular FS values end of the treatment and after treatment increased significantly (p=0.04 and p=0.04). Left ventricular end-diastolic diameter (LVEDd), left ventricular end-systolic diameter (LVESd), interventricular septal thickness at diastole / left ventricular posterior wall end-diastole (IVSd/LVPWd) ratio did not differ significantly between the groups (p>0,05) (Table 3).

**Table 3:** Comparison of the left ventricular systolic and diastolic function before and after the ACTH treatment ACTH - adrenocorticotropic hormone; IVSd - interventricular septal thickness at diastole; IVSs - interventricular septal thickness at systole; LVEDd - left ventricular end-diastolic diameter; LVESd - left ventricular end-systolic diameter; LVPWd -  left ventricular posterior wall end-diastole; FS - fractional shortening; LVd - left ventricule diastole; E - peak modal velocity in early diastole; A - peak modal velocity in late diastole; E/A - early-to-late atrial filling velocity ratio p< 0.05 considered to be statistically significant p^1^: before and the second month of treatment. p^2^: before and the fourth month of treatment. p^3^: the second and the fourth month of treatment.

M-mode echocardiography parameters	Before treatment	The end of treatment (the 2^nd^ month)	After treatment (the 4^th^ month)	p^1^	p^2^	p^3^
IVSd (mm)	5.2 ± 0.20	6.2 ± 0.20	6.9 ± 0.31	<0.01	<0.01	>0.05
IVSs (mm)	5.7 ± 0.30	7.7 ± 0.44	7.6 ± 0.42	<0.01	<0.01	>0.05
IVSd/LVPWd	0.91 ± 0.86	0.89 ± 0.06	1.0 ± 0.13	0.35	0.34	0.35
LVEDd (mm)	20.1 ± 0.62	19.4 ± 0.68	20.4 ± 1.0	0.19	0.18	0.20
LVESd (mm)	12.7 ± 0.54	11.4 ± 0.36	12.1 ± 0.69	0.16	0.17	0.17
LVPWD (mm)	5.5 ± 0.19	6.7 ± 0.40	7.0 ± 0.37	<0.01	<0.01	>0.05
FS (%)	36.0 ± 1.5	41.1 ± 1.9	40.4 ± 1.6	0.04	0.04	>0.05
LVd mass (g)	8.4 ± 0.85	15.7 ± 2.1	20.3 ± 2.5	<0.01	<0.01	>0.05
LVd mass index (g/m^2^)	21.3 ± 1.9	37.6 ± 4.4	46.1 ± 5.1	<0.01	<0.01	>0.05
Mitral valve pulsed wave (PW) Doppler parameters						
E (cm/sec)	1.0 ± 0.05	1.0 ± 0.04	0.97 ± 0.04	0.07	0.07	0.06
A (cm/sec)	0.75 ± 0.06	0.71 ± 0.04	0.67 ± 0.04	0.44	0.45	0.47
E/A	1.5 ± 0.11	1.5 ± 0.11	1.5 ± 0.10	0.85	0.86	0.86

LVPWd before treatment was identified to be significantly increased compared to values at the end of treatment (p<0.01). Similarly, the difference in comparison of LVPWd values before treatment with those after treatment values were significant (p<0.01) (Table 3). The mean left ventricule diastole (LVd) mass and LVd mass index of patients at the end and after treatment were identified to be significantly increased compared to values before treatment (p<0.01 and p<0.01) (Table 3).

All TDI values obtained from mitral lateral annuli before treatment were not different from those data after treatment (p>0.05). Also, the E′ and A′ values were lower on the mitral lateral annuli at the end and after treatment group than the pretreatment group. However, no significant differences were observed between the groups (p>0.05) (Table 4 ).

**Table 4:** Comparison of the tissue Doppler imaging (TDI) parameters between the groups before and after the ACTH treatment ACTH - adrenocorticotropic hormone; E - peak early diastolic velocity; A - peak late diastolic velocity; S - longitudinal peak annular velocities during systole; MPI - myocardial performance index; IVRT - isovolumic relaxation time; IVCT - isovolumic contraction time; MLA - mitral leaflet anterior p< 0.05 is considered statistically significant p^1^: before and the second month of treatment. p^2^: before and the fourth month of treatment. p^3^: the second and fourth of treatment.

MLA TDI parameters	Before treatment	During treatment (the 2^nd^ month)	After treatment (the 4^th^ month)	p^1^	p^2^	p^3^
E′ (cm/sec)	13.4 ± 0.97	13.2 ± 0.98	11.7 ± 0.63	0.31	0.33	0.32
A′ (cm/sec)	8.0 ± 0.41	7.8 ± 0.68	6.9 ± 0.67	0.28	0.25	0.26
S′ (cm/sec)	9.6 ± 0.38	10.5 ± 0.74	10.2 ± 0.35	0.34	0.31	0.38
E′/A′	1.7 ± 0.07	1.7 ± 0.10	1.9 ± 0.18	0.22	0.23	0.20
IVRT (ms)	92.1 ± 11.3	72.6 ± 5.3	91.8 ± 10.2	0.26	0.23	0.24
IVCT (ms)	59.3 ± 6.3	53.2 ± 3.4	63.5 ± 7.9	0.33	0.33	0.33
MPI (%)	0.32 ± 0.01	0.35 ± 0.02	0.36 ± 0.03	0.55	0.53	0.56
E/E′	8.4 ± 0.60	8.3 ± 0.70	8.7 ± 0.79	0.90	0.88	0.91

## Discussion

In the current study, we showed that low dose and short duration ACTH treatment in IS patients has a significant effect on IVSd and LVd mass index. A few studies have investigated the effects of ACTH and steroids in children and adults in the literature. Bobele et al. demonstrated septal hypertrophy with left ventricle posterior wall thickness and interventricular septum ratio >2.0 in five IS patients. In these five cases, the systolic anterior motion of the mitral valve observed during systole without left ventricle outflow tract obstruction. These data are in concordance with our findings, but contrary to our study, they identified concentric hypertrophy in eight patients, which was associated with systemic blood pressure elevation [7]. 

In our study, when heart rates were compared within the different treatment periods, there was a statistically significant decrease identified. Hattori et al. also reported that heart rate decreases during ACTH treatment in 15 patients with a mean age of 9.9 months (range of ages: 5.4 to 23.4 months). ACTH enhances parasympathetic function and causes bradycardia, but these findings are no longer evident one week after the cessation of treatment [8]. Corticosteroids have several cardiovascular side effects that are more common in adults than in children. Corticosteroid related bradycardia is rarely seen as a side effect of steroids, and it is mostly related to intravenous steroid treatment. Although adult patients presenting with bradycardia following oral corticosteroid use have been reported, children with bradycardia have rarely been reported previously [4]. Different from our study, Pishgahi et al. identified a statistically significant increase in heart rate after pulse steroid treatment [9]. Our findings show that low dose and short duration ACTH is an effective and relatively safe treatment for patients with IS.

The surface ECGs of the patients in our study did not show any significant arrhythmia. However, we did not perform 24-hour rhythm Holter monitoring to assess the rhythm disturbances during a day. As there are very few studies of children in the literature, most of the data obtained from adult studies. Vasheghani-Farahani et al. identified most commonly sinus tachycardia in their study, but also observed a variety of other arrhythmias. Generally, they indicate that these effects observed at high doses of treatment [10].

The expected effects of steroids and ACTH on blood pressure is hypertension. Mundell et al. reported that hypertension could be seen in 14% of their patients during ACTH treatment [11]. In our study, we observed that there was no significant difference in systolic and diastolic blood pressures. Similar to our findings, in the literature, there are studies with no significant increase in systolic and diastolic blood pressure parameters [9].

TDI is more sensitive than standard echocardiography in detecting diastolic dysfunction. The investigators reported that the mitral E'-value was significantly decreasing, the E/E' rate was increased, and the MPI was increased in the patients with diastolic dysfunction due to different systemic illness [6, 12]. 

The MPI is a sensitive noninvasive Doppler parameter that can be used to assess global systolic and diastolic myocardial functions at the same time [12]. In our study, after ACTH treatment, there was an increase in LV MPI and a decrease in E' identified; however, these did not reach statistical significance. When the general literature is investigated, there are few studies reporting the effect of steroids on heart muscle using tissue Doppler echocardiographic methods. Baykan et al. reported diastolic dysfunction in Cushing syndrome using TDI [13]. In their study, E'-value significantly decreased, the E/E' rate was increased, and the MPI was increased in the patients compared to the control group. Also, in this study, they reported an increase in IVRT and the left ventricle index. Serum cortisol level was reported to be directly proportional to MPI and E/E' ratio and inversely proportional to E' and S'. Muiesan et al. reported a decrease in E and E/A ratios in 42 patients with Cushing syndrome. Furthermore, they determined the left ventricle hypertrophy and concentric hypertrophy in the study group [14]. This thickening was considered because of the direct effect of cortisol on the heart muscle. In our study, the statistical insignificance of LVPWd parameters may be due to the low-dose and short-duration of steroid use.

In addition to the strong aspects, the current study had several limitations. First, a relatively small number of patients in a single-center were studied, which may have affected the power of the study. Second, all ECG and echocardiographic studies were performed by a single observer in a blinded fashion, but intraobserver reliability was not assessed. Third, although the ECGs of the patients were normal, 24-hour rhythm Holter monitoring was not performed to assess the rhythm during a day. Lastly, the short follow-up duration is one of the limitations of the study.

## Conclusions

We showed mild septal hypertrophy and an increase in the left ventricle mass index with ACTH treatment. This may be related to the low ACTH dose and short duration of the treatment. Septal hypertrophy did not show progression after treatment; however, longer follow-up durations should be required to evaluate the changes in the heart muscle. It is important that patients are monitored by a pediatric cardiologist with a multidisciplinary approach in terms of identifying efficacy and complications of treatment. Further studies are needed to show the effect of ACTH treatment on cardiac function.
